# Paving the Way to Food Grade Analytical Chemistry: Use of a Natural Deep Eutectic Solvent to Determine Total Hydroxytyrosol and Tyrosol in Extra Virgin Olive Oils

**DOI:** 10.3390/foods10030677

**Published:** 2021-03-22

**Authors:** Vito Michele Paradiso, Francesco Longobardi, Stefania Fortunato, Pasqua Rotondi, Maria Bellumori, Lorenzo Cecchi, Pinalysa Cosma, Nadia Mulinacci, Francesco Caponio

**Affiliations:** 1 Department of Biological and Environmental Sciences and Technologies, University of Salento, Centro Ecotekne, S.P. 6 Lecce-Monteroni, I-73100 Lecce, Italy; 2 Department of Soil, Plant and Food Sciences, University of Bari, Via Amendola 165/a, I-70126 Bari, Italy; stefania.fortunato@uniba.it (S.F.); francesco.caponio@uniba.it (F.C.); 3 Department of Chemistry, University of Bari, Via Orabona 4, I-70126 Bari, Italy; pattyrotondi83@alice.it (P.R.); pinalysa.cosma@uniba.it (P.C.); 4 Department of NEUROFARBA, and Multidisciplinary Centre of Research on Food Sciences (M.C.R.F.S.-Ce.R.A.), University of Firenze, Via Ugo Schiff 6, I-50019 Sesto F.no (Firenze), Italy; maria.bellumori@unifi.it (M.B.); lo.cecchi@unifi.it (L.C.); nadia.mulinacci@unifi.it (N.M.)

**Keywords:** phenolic compounds, acidic hydrolysis, derivative UV spectroscopy, green chemistry, screening methods, health claim

## Abstract

Extra virgin olive oil (EVOO) is well known for containing relevant amounts of healthy phenolic compounds. The European Food Safety Authority (EFSA) allowed a health claim for labelling olive oils containing a minimum amount of hydroxytyrosol (OHTyr) and its derivatives, including tyrosol (Tyr). Therefore, harmonized and standardized analytical protocols are required in support of an effective application of the health claim. Acid hydrolysis performed after extraction and before chromatographic analysis has been shown to be a feasible approach. Nevertheless, other fast, green, and easy methods could be useful for on-site screening and monitoring applications. In the present research, a natural deep eutectic solvent (NADES) composed of lactic acid and glucose was used to perform a liquid/liquid extraction on EVOO samples, followed by UV-spectrophotometric analysis. The spectral features of the extracts were related with the content of total OHTyr and Tyr, determined by the acid hydrolysis method. The second derivative of spectra allowed focusing on three single wavelengths (i.e., 299 nm, 290 nm, and 282 nm) significantly related with total OHTyr, total Tyr, and their sum, respectively. In particular, the sum of OHTyr and Tyr could be determined with a root mean square error of prediction of 29.5 mg kg^−1^, while the limits of quantitation and detection were respectively 11.8 and 4.9 mg kg^−1^. The proposed method, therefore, represents an easy screening tool, with the use of a green, food-derived solvent, and could be considered as an attempt to pave the way for food grade analytical chemistry.

## 1. Introduction

The health properties of phenolic compounds contained in extra virgin olive oil (EVOO) have been clearly established by many scientific papers [1,2,3]. On this basis, according to the European Food Safety Authority (EFSA), since 2012 European regulation has allowed the use of a health claim in olive oil labeling [4]. The health claim states as follows: “Olive oil polyphenols contribute to the protection of blood lipids from oxidative stress” and can be applied to olive oils containing at least 5 mg of hydroxytyrosol (OHTyr) and its derivatives (e.g., oleuropein complex and tyrosol) per 20 g of product.

Based on this regulation, scholars focused their attention on analytical issues related to the assessment of the suitability of oils to be labelled with the health claim. Several analytical methods have been proposed, usually comprising an extraction step followed by separation by means of liquid chromatography, gas chromatography, or capillary electrophoresis, and a suitable detection method. 

Irrespective of the separation and detection methods, the quantification step of the target compounds remains a critical issue. In fact, OHTyr and tyrosol (Tyr) are present in EVOO in their free form, as well as esterified in several derivatives [5]. Therefore, the quantitation of each derivative is difficult to obtain. A widely adopted approach to overcome this difficulty is to carry out a hydrolysis step prior to separation and analysis, in order to quantify, as free forms, the total of free and linked OHTyr and Tyr. This approach has been adopted either directly on the oil or on the phenolic extract, with quite satisfactory performances [6,7,8,9]. 

Nevertheless, the adoption of separation methods for the analytical determination, though being affordable and sensitive, requires expensive equipment, toxic/pollutant reagents, and trained operators. The availability of easy, less expensive, and operator- and environment-friendly methods, rather than being an alternative, could be a valid analytical complement, useful for screening purposes, even in oil mills or bottling plants as a quality monitoring or a decision supporting tool [10]. Some efforts in this direction have been made. The Folin–Ciocalteu assay, a widely used spectrophotometric method, has provided good preliminary results [11]. 

In addition, natural deep eutectic solvents (NADES) have been proposed as solvents for easy screening methods. NADES are green solvents consisting of mixtures of one or more hydrogen bond acceptor–donor pair that, in appropriate molar ratios, generate strong intermolecular interactions [12]. Compared to deep eutectic solvents (DES), NADES are obtained from molecules naturally present in living organisms as metabolites [13]. They are being increasingly used for analytical purposes [12,14]. NADES have been successfully applied in the analysis of EVOO phenolic compounds as extraction media prior to liquid chromatography [15], electrochemical analysis [16], and spectrophotometric analysis [10,17]. In one case, NADES extraction and direct spectrophotometric analysis allowed assessing the amount of OHTyr and Tyr derivatives determined as the sum of the free and linked forms determined by high-performance liquid chromatography (HPLC), thus providing a useful tool to label EVOO according to the EU health claim [10]. 

In view of a better harmonization of the green screening methods with the candidate official methods related to the health claim, there is still the need for easy and green methods allowing assessing total OHTyr and Tyr as free forms, as determined by emerging hydrolysis methods. The present research was therefore aimed at setting up the spectrophotometric determination of total OHTyr and Tyr, free and linked, after extraction with a NADES composed of lactic acid, glucose, and water.

## 2. Materials and Methods

### 2.1. Reagents and Oil Samples

Glucose (≥99.5%), lactic acid (90%), formic acid, sulfuric acid (95.0–98.0%), methanol and acetonitrile of HPLC grade, methanol and ethanol of analytical grade, hydroxytyrosol, and tyrosol were purchased from Sigma-Aldrich (Sigma-Aldrich Co. LLC, St. Louis, MO, USA). Ultrapure water was obtained from an Elga Purelab Option R system (Veolia Environnement S.A., Paris, France). Extra virgin olive oil (EVOO) samples (*n* = 26) were obtained from producers and research laboratories. They differed by geographical origin (different Italian regions), cultivar, olive maturity, and extraction technology.

### 2.2. NADES Preparation

The NADES was obtained according to a previous work, with slight modifications [17]. Lactic acid, glucose, and water (5:1:3 molar ratio) were mixed by means of a magnetic stirrer at 50 °C for about 90 min, until obtaining a clear solution. Further dilution of the components in such molar ratio was carried out with 20% (*v*/*v*) water to reduce solvent viscosity. Previous studies reported the possibility of using water to tailor the solvent properties, mainly viscosity [18,19]. In particular, Pisano et al. proved that the same NADES used in this study held its supramolecular structure throughout dilutions [19].

### 2.3. Extraction with NADES and Spectrophotometric Analysis of the Extract (NADES-UV Method)

The EVOO sample (0.5 g) was submitted to extraction with 5 mL of NADES. After intense agitation with a vortex (5 min), centrifugation was performed for 10 min at 6000 rpm. The lower layer (NADES plus phenolics) was recovered, centrifuged again at 9000 rpm for 5 min and, after being recovered, finally filtered at 0.45 μm using nylon filters (VWR International, Center Valley, PA, USA).

The NADES extracts were analyzed by UV-Vis spectrophotometry in the wavelength range 250–400 nm by means of an Agilent Cary 60 spectrophotometer (Agilent Technologies, Santa Clara, CA, USA). The acquisition parameters were the following: 1 cm optical path, 2 nm slit, and 60 nm min^−1^ scan rate. Pure NADES was used for baseline correction. For quantitation purposes, calibration curves of OHTyr and Tyr in NADES were built in the ranges 3–30 mg L^−1^ and 5–40 mg L^−1^, and at wavelengths 299 nm and 290 nm, respectively. A total phenolic calibration curve was also built at 282 nm in the range of total 8–70 mg L^−1^ by using mixtures of OHTyr and Tyr, and employing for each compound the same concentration range reported above.

### 2.4. HPLC Analysis of Total Phenolic Compounds Free OHTyr and Tyr, and Total OHTyr and Tyr 

The analysis of free OHTyr and Tyr and of total phenolic compounds was performed according to the IOC official method [20]. Phenolic compounds were extracted from the EVOO sample (2.0 g) with 5 mL MeOH:H_2_O 80:20 *v*/*v* by shaking for 1 min, extracting in an ultrasonic bath for 15 min, and centrifuging at 5000 rpm for 25 min. The supernatant was filtered on PVDC filters (0.45 μm) and analyzed by HPLC. An HP 1100 system coupled to a diode array detector (Agilent Technologies, Santa Clara, CA, USA) was used. The column was a SphereClone ODS-2, 5 µm, 250 × 4.6 mm id kept at room temperature during chromatographic separation. The eluents were formic acid solution (pH 3.2), acetonitrile, and methanol. The analyses were carried out at a flow rate of 1 mL min^−1^, with an injection volume of 20 µL and a total analysis time of 82 min, applying the gradient reported in the IOC method. The areas were registered at 280 nm, with syringic acid as the internal standard. The content of phenolic compounds was expressed as mg of Tyr per kg of oil.

Total OHTyr and Tyr were determined by HPLC after acid hydrolysis (with sulfuric acid at 80 °C for 2 h) of the hydroalcoholic extract obtained according to the IOC method [21]. For HPLC analysis of the hydrolyzed extracts, formic acid solution at pH 3.2 (solvent A) and acetonitrile (solvent B) were used as eluents. An HP1200 liquid chromatograph coupled to a diode array detector (Agilent Technologies, Santa Clara, CA, USA) was used with a 150 × 3 mm (5 µm) Gemini RP18 column (Phenomenex, Torrance, CA, USA). The flow rate and the injection volume were 0.4 mL min^−1^ and 20 µL, respectively. A linear gradient was applied starting from 95% A to 70% A in 5 min, to 50% A in 5 min, to 2% A in 5 min with a final plateau of 5 min. Total analysis time was 22 min. For UV detection, the wavelength of 280 nm was used. The results were expressed as mg of Tyr per kg of oil for tyrosol, and as mg of OHTyr per kg of oil for hydroxytyrosol after application of the formula reported by Bellumori et al. [22], for keeping into account the 35% overestimation of OHTyr when the calibration curve of tyrosol is used. 

### 2.5. NADES-UV Method Validation

The NADES-UV method was validated by measuring the linearity, the limits of detection (LOD) and quantitation (LOQ), as well as the precision (repeatability), and accuracy (recovery). Linearity of the response in the analytical range was assessed by regression analysis and R^2^ values. LOD was expressed as 3.3 × S.D. of the blank (*n* = 3), while LOQ was expressed as 10 × S.D. of the blank (*n* = 3). Sunflower oil was used as blank sample. Repeatability was assessed with repeated intra-day (*n* = 3 × 5 samples) and inter-day (*n* = 3 × 1 sample × 3 days) trials. Recovery was determined as apparent recovery compared to the reference method [21,22] and expressed in percent.

### 2.6. Statistical Analysis

Spectra, after subtraction of the pure solvent spectrum, were pre-processed using Solo 8.6.2 (Eigenvector Research, Inc., Manson, WA, USA), by applying the second derivative Savitzky–Golay algorithm (polynomial order: 2, window: 11 pt). Analysis of correlation, least squares regression, and analysis of variance (ANOVA) were performed with Origin 2021 (OriginLab Corporation, Northampton, MA, USA).

## 3. Results and Discussion

### 3.1. Spectral Characterization of NADES Standard Solutions

**Figure 1:**
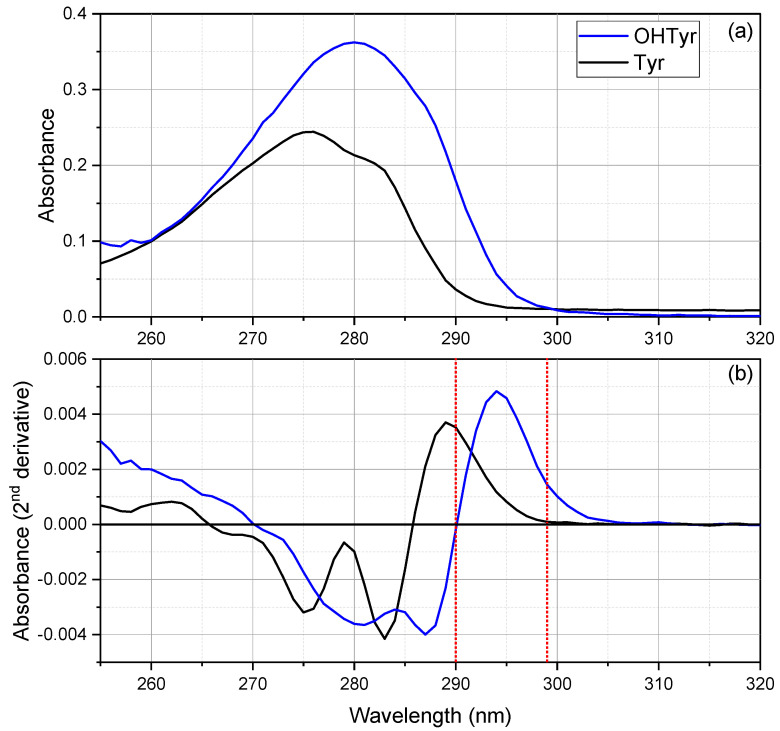
Mean spectra (**a**), and second derivatives (**b**) of OHTyr and Tyr solutions in natural deep eutectic solvent (NADES) (20 mg L^−1^, *n* = 3). The red dotted reference lines correspond to the wavelengths selected for the calibration curves.

The spectra of OHTyr and Tyr in NADES were acquired and analyzed. Figure 1 reports the mean spectra (a), and the second derivatives (b) of both compounds at the concentration of 20 mg L^−1^. The application of a second derivative treatment allows highlighting minor or subtle spectral features, and resolves spectral overlapping [23,24,25]. Valleys in the second derivative spectra correspond to peaks in direct absorption spectra, and vice versa.

As regards OHTyr, the spectrum showed a large peak at 280 nm (λ_max_ = 280 nm, ε_λmax_ = 2793 M^−1^ cm^−1^). The molar extinction (e) determined for OHTyr in NADES was lower than that reported for OHTyr in water [26]. The second derivative showed more spectral features, with two negative peaks at 281 nm and 287 nm, and a positive peak at 294 nm, with a slight shoulder on the right. Spectra of NADES solutions at different concentrations of OHTyr (3, 5, 10, 20, 30 mg L^−1^) were acquired. Then, a rapid screening via analysis of correlation (data not shown) indicated that the wavelengths that showed the best correlation of the second derivative of the spectrum with the concentrations of OHTyr were 294, 299, 286, and 280 nm (*r* = 0.99878, *r* = 0.9986, *r* = 0.99853, and *r* = 0.99839, respectively, *n =* 15). 

As regards Tyr, the spectrum showed a peak at 276 nm and a shoulder at about 283 nm (λ_max_ = 276 nm, ε_λmax_ = 1676 M^−1^ cm^−1^). Therefore, hypsochromic and hypochromic shifts of the absorbance were observed for the monophenolic group of Tyr compared to the *o*-diphenolic structure of OHTyr. Spectra of NADES solutions at different concentrations of Tyr (5, 10, 20, 30, 40 mg L^−1^) were acquired. The analysis of correlation (data not shown) indicated that the wavelengths that showed the best correlation of the second derivative of the spectrum with the concentrations of Tyr were 290, 274, and 282 nm (*r* = 0.99911, *r* = 0.99897, *r* = 0.99883 respectively, *n =* 15). 

### 3.2. NADES Extraction and Extracts Characterization

**Figure 2:**
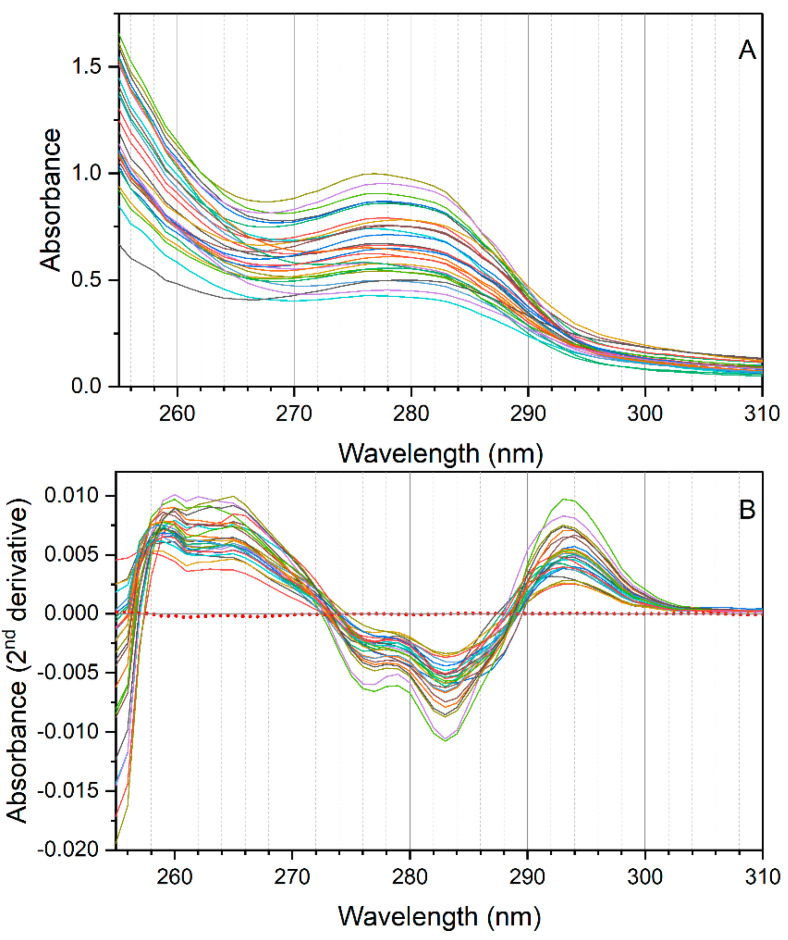
Spectra (**A**), and second derivatives of spectra (**B**) of the NADES extracts of the EVOO samples (*n* = 26). The red dotted line in (**B**) reports the second derivative of the NADES extract of a sample of sunflower oil.

A sample set of EVOOs (*n* = 26) was submitted to NADES extraction. Figure 2 reports the spectra of the NADES extracts (A), as well as the second derivatives (B) of the spectra. As a blank sample, a sunflower oil was submitted to NADES extraction in order to assess possible matrix effects due to components other than phenolic compounds, of which sunflower oil is void. The red dotted line in the panel of the second derivative spectra (B) represents the second derivative spectrum of the NADES extract obtained from the sample of sunflower oil (mean of three replicates). As can be seen, the curve appears flat and very near to zero. The second derivative spectra of the NADES extracts clearly reflect the spectral features of the mono- and *o-*diphenolic structures observed in the spectra of the standard compounds. In particular, negative peaks at 276–277 nm and 283 nm could be observed in all samples, as well as a positive peak at 292–294 nm. These wavelengths proved relevant in the analysis of EVOO phenolic compounds via NADES extraction [10,17].

### 3.3. Analysis of Correlation

**Figure 3:**
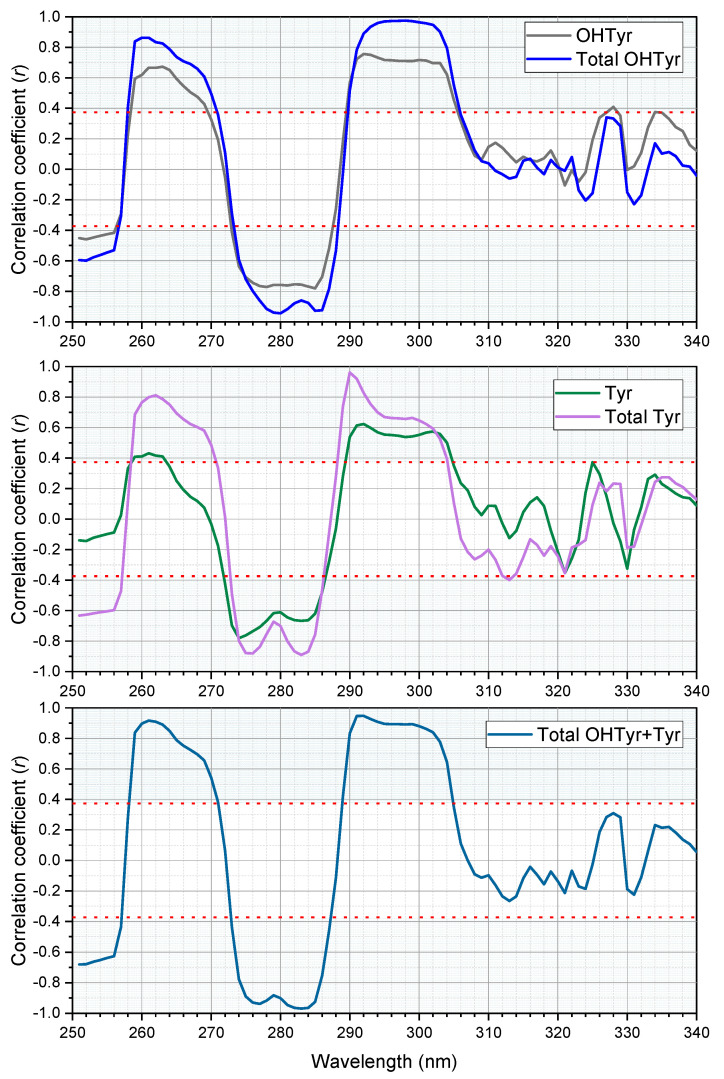
Correlation plots of the second derivatives of absorbance of the NADES extracts (*n* = 26) versus the content of free (before hydrolysis) and total (after hydrolysis) OHTyr and Tyr, and their sum. The red dotted lines and the light blue areas indicate the thresholds of significance (*p* = 0.05).

An analysis of correlation was carried out on the second derivative of absorption of the NADES extracts of the 26 EVOO samples with their contents of free and total OHTyr and Tyr (reported in Supplementary Table S1). The correlation analysis confirmed the relationships between the spectral features of the extracts and the content of phenolic compounds in the oils (Figure 3). OHTyr contents were associated with a peak of negative correlation with the second derivative of absorption at 280 and 285 nm. On the other hand, a positive correlation was observed with the second derivative of absorption in the range 293–302 nm (maximum at 299 nm, where a *r* value of 0.9563 was reached). Hypsochromic shifts were observed for the peaks of correlation with the contents of Tyr (negative correlation with the second derivatives at 276 and 283 nm, and a maximum positive correlation at 290 nm). These results confirm that the spectral information contained in the NADES extracts of the oils was strictly related to their content of OHTyr and Tyr.

**Figure 4:**
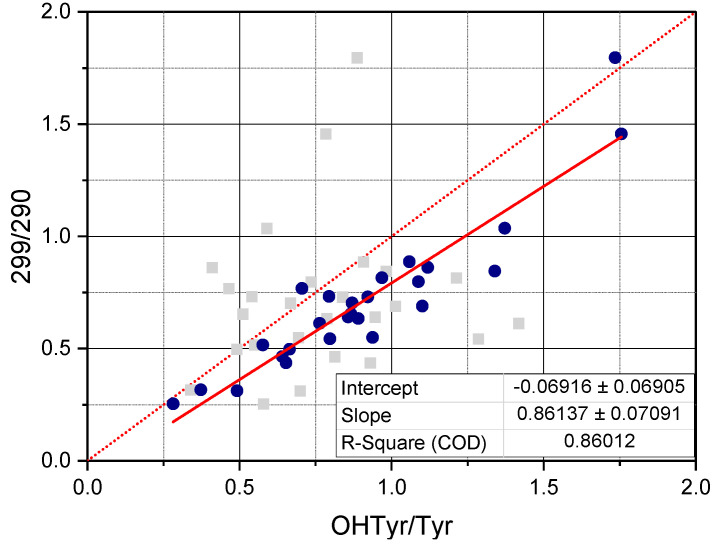
The scatter plot reports the ratio, 299/290, between the second derivative of the absorbance of NADES extracts at 299 nm and the second derivative of the absorbance of NADES extracts at 290 nm versus the ratio OHTyr/Tyr between the content of OHTyr and the content of Tyr, both free (grey scatter) and total (blue scatter). The second derivatives at 299 and 290 have been normalized by the molar extinction coefficient of respectively OHTyr and Tyr. The box reports the parameters of the linear regression of the 299/290 ratio versus the total OHTyr/total Tyr ratio.

Notably, correlations were clearly higher when relating total OHTyr and Tyr rather than their free forms. This means that absorbance of the NADES extracts in the UV range can be attributed, rather than to specific molecules (i.e., free OHTyr and free Tyr), to specific moieties of a wide range of phenolic antioxidants (i.e., both free and esterified OHTyr and Tyr moieties). This is confirmed by the data in Figure 4, were the ratio between the values of the second derivative of absorbance at 299 nm and 290 nm (normalized by the extinction coefficients respectively of OHTyr and Tyr) of the NADES extracts is plotted against the corresponding ratio of either free OHTyr to free Tyr, or total OHTyr to total Tyr.

### 3.4. Determination of Total OHTyr and Tyr

On the basis of the UV spectral information of the NADES extracts, a quantitation of total OHTyr and Tyr could be approached. According to the analysis of OHTyr and Tyr spectra, as well as to the correlation analysis of the NADES extracts of the EVOO sample set, two different wavelengths were selected for the determination of total OHTyr and Tyr. As regards OHTyr, considering the overlaps with the spectra of Tyr, the wavelength selected for the calibration curve was 299 nm. For Tyr, the wavelength 290 nm was considered optimal, since it corresponded with a zero-point of the derivative of OHTyr spectrum.

The selected wavelengths allowed obtaining calibration curves of both OHTyr and Tyr in NADES, in the ranges 3–30 mg L^−1^ for OHTyr, and 5–40 mg L^−1^ for Tyr, with R^2^ values of 0.997 and 0.998, respectively.

Table 1 reports the parameters of the calibration curves:

*A_λ_* = *a* + *b* · *C (*mg L^−1^*)*, (1)

where *A_λ_* is the second derivative of the absorbance at the selected wavelength l, *C* is the concentration of the analyte in NADES, *a* is the intercept, and *b* is the slope.

**Figure 5:**
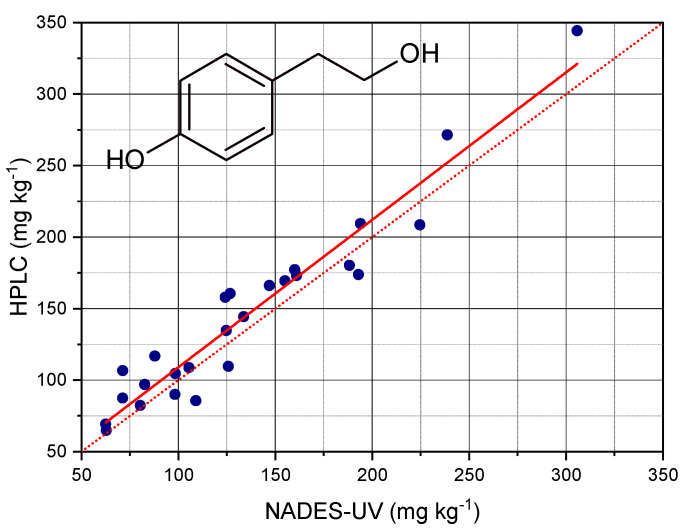
**C**ontents of total Tyr determined by HPLC after hydrolysis of the extracts plotted versus the contents of total Tyr determined by NADES extraction and UV analysis. The red dotted line has the function y = x, the red solid line is the fitted regression.

The determination of Tyr was carried out on the basis of the second derivative of absorption at 290 nm of the extracts and of the parameters of the calibration curve of Tyr in NADES. Figure 5 reports the contents of total Tyr determined by HPLC after hydrolysis of the extracts plotted versus the contents of total Tyr determined by NADES extraction and UV analysis. 

The analysis of regression showed that the contents of Tyr determined by the NADES-UV method allowed predicting the contents determined by acid hydrolysis and HPLC with an accuracy corresponding to a R^2^ value of 0.925. The parameters of the regression are reported in Table 2. The prediction performance of the method was clearly better compared to those observed in our previous works [10,17]. The root mean square error (RMSE) was 18 mg kg^−1^, corresponding to a RMSE% of 5.8%. The improved performance of this method could be due to the use of derivative processing of the spectra. Moreover, the comparison with the method of determination of total Tyr after hydrolysis could highlight the sensitivity of the NADES-UV method towards specific structural features in the phenolic pattern of EVOO. The slope of the curve was very near to 1; the amounts determined nu NADES- UV were very close to those determined by acid hydrolysis and HPLC analysis.

**Figure 6:**
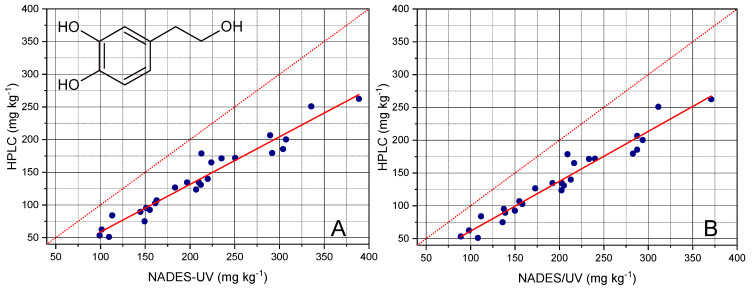
**C**ontents of total OHTyr determined by HPLC after hydrolysis of the extracts plotted versus the contents of total OHTyr determined by NADES extraction and UV analysis. The red dotted line has the function y = x, the red solid line is the fitted regression. The panel on the left (**A**) plots the values of total OHTyr determined prior the correction of the second derivative of absorbance at 299 nm, while the panel on the right (**B**) plots the values obtained after the correction (please see text for details on the correction procedure).

The quantitation of OHTyr provided the results reported in Figure 6 and Table 3. The analysis of regression showed the possibility of predicting the content of total OHTyr in EVOO with an accuracy corresponding to a *R*^2^ value of 0.942, a RMSE of 14 mg kg^−1^, and a RMSE% of 5.0%. It should be noted that the NADES-UV method provided an overestimation of total OHTyr, as showed by the slope value of about 0.73. However, the good fitting performance allowed satisfactorily correcting the obtained value and predicting the result of the HPLC analysis. 

A possible explanation for such overestimation could be related to the matrix effects attributable to the phenolic pattern of EVOO; the NADES-UV method implies the direct analysis of the extract, without chromatographic separation. Therefore, a possible interference of Tyr, as well as possible differences in absorptivity of the esterified forms with respect to free forms could have occurred. This hypothesis seems to be confirmed by the fact that total Tyr was also correlated with the 299 nm wavelength (*r* = 0.95); this would indicate a contribution of Tyr to the absorbance at that wavelength. 

Therefore, in order to reduce the total Tyr contribution, the values of the second derivative at 299 nm were corrected as follows, prior to applying the parameters of the calibration curve of OHTyr: A calibration curve for Tyr was built at 299 nm; then, the contents of Tyr determined using the calibration curve built at 290 nm were applied to calculate the corresponding value of the second derivative at 299 nm; the value calculated was then subtracted to the value measured; the difference was finally used to determine the content of OHTyr. 

As shown in Figure 6B, even if a slight decrease of the overestimation and a contemporary slight improvement of the fitting was obtained after correction, a not negligible overestimation of OHTyr was still observed, suggesting the hypothesis of a matrix effect as the basis of the overestimation of OHTyr.

### 3.5. Sum of OHTyr and Tyr

An attempt to determine the sum of total OHTyr and Tyr was made by building a calibration curve with mixtures of OHTyr and Tyr. OHTyr and Tyr were solubilized in te NADES in different concentrations, with a total range of 8 to 70 mg L^−1^. Analysis of correlation (data not shown) indicated 282 nm as the wavelength with the highest correlation with the content of total OHTyr + Tyr. This agrees with the results of the correlation analysis of the spectra of the NADES extracts with the contents of OHTyr and Tyr in EVOOs. The calibration curve built with the second derivative of the absorption at 282 nm as a function of total OHTyr + Tyr showed the parameters reported in Table 4.

**Figure 7:**
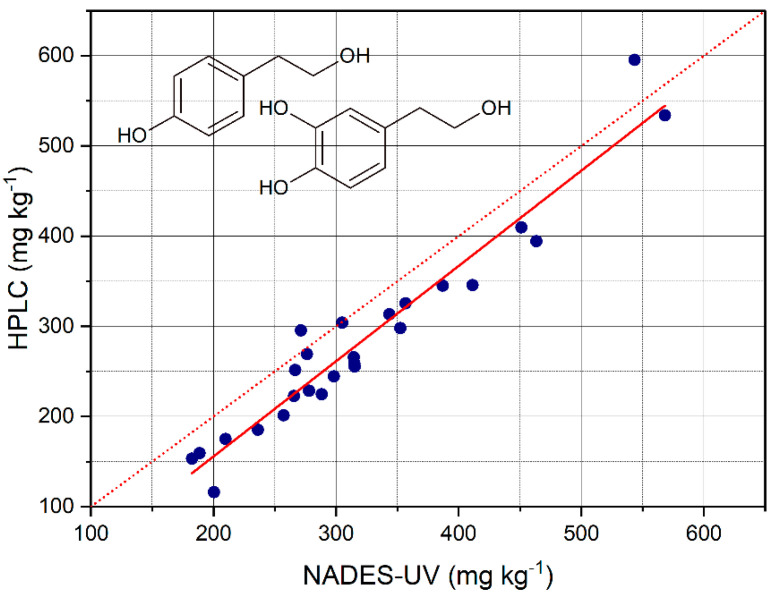
**C**ontents of total OHTyr + Tyr determined by HPLC after hydrolysis of the extracts plotted versus the contents of total OHTyr + Tyr determined by NADES extraction and UV analysis. The red dotted line has the function y = x, the red solid line is the fitted regression.

The total content of OHTyr + Tyr in the EVOO samples was measured based on the calibration curve and on the second derivative of the absorption at 282 of the NADES extracts. The values determined were plotted against the values obtained by HPLC after acid hydrolysis of the methanolic extracts, and a regression analysis was carried out. The results are reported in Figure 7 and Table 5. The contents of OHTyr + Tyr were, also in this case, overestimated with respect to the method by HPLC, with a mean residual of 37 mg L^−1^. The mean recovery was 117 ± 15%. The R^2^ value of the regression was 0.931, confirming a satisfactory predictive capacity of the NADES-UV method with respect to the method by HPLC. The overestimation was confirmed by the regression analysis with the significance of the intercept. On the other hand, the slope of the regression was very close to 1.

Therefore, the wavelength 282 nm resulted in a common wavelength of absorption of both OHTyr and Tyr, unlike 299 nm and 290 nm that were specific spectral features. The values of RMSE and RMSE% were 29.5 mg kg^−1^ and 5.6%, respectively. Compared to the performance of SVM regression applied to the whole spectra of NADES extract to determine the sum of OHTyr and Tyr derivatives assessed by HPLC, as reported in our previous work [10], the RMSE of prediction resulted lower (29.5 vs. 35.5 mg kg^−1^), while it reached even higher levels when considering the single wavelength of 283 nm (58.7 mg kg^−1^). As regards the R^2^ value, it increased to 0.931 from the 0.84 and 0.64 of SVM and linear regression, respectively, of our previous work.

Table 6 reports the figures of merit of the method. The LOD and LOQ were respectively 3.9 and 11.8 mg kg^−1^. As regards repeatability, the CV% of the method remained around or below 5%.

The proposed analytical approach can be considered as a screening method applicable to assess the levels of total OHTyr and Tyr, as determined by the emerging hydrolysis methods [6]. Some key aspects could support their use as a tool with complementary functions to the more accurate and precise chromatographic methods:

- Use of environment- and operator-friendly solvents;
- Use of low-cost equipment;
- Easy analytical procedure;
- Short analysis time.

Due to these features, this analytical method could be applied to the field (e.g., oil mills, bottling plants, storage plants) and allow the monitoring of the levels of phenolic compounds related to the health claim. A continuous monitoring of this parameter and its trends during storage before bottling, as well as after bottling, could make operators aware of the evolving quality of individual EVOO batches, and therefore more confident in the use of the health claim on labels [27,28]. The appropriate adoption of the health claim on labels could also support focused communication and be considered for possible its effects on consumer perception and choices and the segment the trade category of EVOO, with eventual benefits for the EVOO value chain [29,30]. 

To the best of our knowledge, there are few alternative screening methods with the same aim. Reboredo-Rodríguez et al. [11] previously showed that the traditional Folin–Ciocalteu assay provided, for a sample set of 12 EVOOs, results comparable to the amounts of total OHTyr and Tyr determined by acid hydrolysis and HPLC. Nevertheless, the performance of this approach, compared to the reference method, was lower (*r* = 0.94). Moreover, the analytical reagents are not environment friendly.

Shabani et al. [16] recently proposed the electrochemical detection of EVOO phenolic compounds extracted with the same NADES used in our previous work [17]. The method proposed by the authors was easy and environment- and user-friendly, though the analytical target includes all the phenolic compounds contained in EVOO and not only those accounting for the health claim.

The method presented in this paper, could provide, therefore, a feasible opportunity for operators of the EVOO chain to access relevant chemical information. The NADES adopted, composed of lactic acid, glucose, and water, could pave the way for a new category of green analytical chemistry: food grade analytical chemistry. It is not simply a paradox: after sustainable reagents, can we move towards edible reagents?

## 4. Conclusions

A green and simple method, using food grade reagents, was set up as an on-site, environment- and operator-friendly tool for screening of olive oils according to the content of total (i.e., free and linked) OHTyr and Tyr. The reagents (glucose, lactic acid, and water) and the equipment (a spectrophotometer) required make this method affordable and feasible in contexts other than analytical labs. The limits of detection and quantitation (3.9 and 11.8 mg kg^−1^) and the repeatability (CV% ≤ 5%) can be considered satisfactory for screening purposes. The results obtained, although leading to a slight overestimation, fit with those obtained by the reference HPLC method, and showed good predictive capacity. The NADES-UV method could be a suitable complement to more complex methods (involving extraction, acid hydrolysis, and HPLC analysis) that seem to be the best approach to address the requirements of the EFSA health claim. The availability of a complete analytical platform comprising both on-site screening methods and accurate laboratory determination could support the value chain operators in EVOO differentiation and segmentation, as well as consumers in gaining increasing awareness.

## Figures and Tables

**Table 1:** Parameters of the calibration curves of OHTyr and Tyr in NADES.

	Tyr	OHTyr
Wavelength of second derivative (λ)	290 nm	299 nm
Intercept (a)	−1.62972 × 10^−4^	−2.87704 × 10^−5^
Slope (b)	1.82318 × 10^−4^	7.11259 × 10^−5^
*R* ^2^	0.998	0.997
Range	5–40 mg L^−1^	3–30 mg L^−1^

**Table 2:** Parameters of the regression analysis of total Tyr determined by NADES-UV versus total Tyr determined by acid hydrolysis and HPLC.

	Value	Significance
Intercept	6.26613	0.49
Slope	1.02911	<0.001
*R* ^2^	0.925	
RMSE	18.0	
RMSE%	5.8	

**Table 3:** Parameters of the regression analysis of total OHTyr determined by NADES-UV, versus total OHTyr determined by acid hydrolysis and HPLC.

	Value	Significance
Before correction		
Intercept	−13.84855	0.10
Slope	0.72584	<0.001
*R* ^2^	0.942	
RMSE	14.0	
RMSE%	5.0	
After correction		
Intercept	−13.48954	0.10
Slope	0.75556	<0.001
*R* ^2^	0.945	
RMSE	13.7	
RMSE%	4.9	

**Table 4:** Parameters of the calibration curve of OHTyr + Tyr in NADES.

	Tyr + OHTyr
Wavelength of second derivative (λ)	282 nm
Intercept (a)	1.30785 × 10^−4^
Slope (b)	−1.80048 × 10^−4^
*R* ^2^	0.995
Ranges	5–40 mg L^−1^ (Tyr); 3–30 mg L^−1^ (OHTyr); 8–70 mg L^−1^ (OHTyr + Tyr)

**Table 5:** Parameters of the regression analysis of total OHTyr + Tyr determined by NADES-UV versus total OHTyr + Tyr determined by acid hydrolysis and HPLC.

	Value	Significance
Intercept	−55.61922	0.00679
Slope	1.05626	<0.001
*R* ^2^	0.931	
RMSE	29.5	
RMSE%	5.6	

**Table 6:** Figures of merit of the method of analysis of total OHTyr + Tyr in EVOOs by NADES extraction and UV analysis of the second derivative spectra.

	OHTyr + Tyr
LOQ (limit of quantification)	11.8 mg kg^−1^
LOD (limit of detection)	3.9 mg kg^−1^
Apparent recovery (%) ^1^	116.8 ± 15.2
Intra-day repeatability (CV%, *n* = 3 × 5 samples)	2.7%
Inter-day repeatability (CV% *n* = 3 × 1 sample × 3 days)	5.1%

^1^ Compared to the amounts determined by the reference method [21].

## Data Availability

The data presented in this study are available on request from the corresponding author.

## Supplementary Materials

The following are available online at https://www.mdpi.com/2304-8158/10/3/677/s1. Table S1: Contents of OHTyr an Tyr in the EVOO samples set, determined by the reference method before and after acid hydrolysis.
